# Advantages and limitations of classic and 3D QSAR approaches in nano-QSAR studies based on biological activity of fullerene derivatives

**DOI:** 10.1007/s11051-016-3564-1

**Published:** 2016-08-29

**Authors:** Karolina Jagiello, Monika Grzonkowska, Marta Swirog, Lucky Ahmed, Bakhtiyor Rasulev, Aggelos Avramopoulos, Manthos G. Papadopoulos, Jerzy Leszczynski, Tomasz Puzyn

**Affiliations:** 1 Laboratory of Environmental Chemometrics, Faculty of Chemistry, Institute for Environmental and Human Health Protection, University of Gdansk, Wita Stwosza 63, 80-308 Gdansk, Poland; 2 Interdisciplinary Nanotoxicity Center, Department of Chemistry and Biochemistry, Jackson State University, 1400 JR Lynch Street, Jackson, MS 39217-0510 USA; 3 Center for Computationally Assisted Science and Technology, North Dakota State University, 1805 NDSU Research Park Drive, Post Office Box 6050, Fargo, ND 58108 USA; 4 Institute of Biology, Pharmaceutical Chemistry and Biotechnology, National Hellenic Research Foundation, 48 Vas. Constantinou Ave., 11635 Athens, Greece

**Keywords:** Nano-QSAR, 3D QSAR, CoMFA, Nanomaterials, Toxicity, Environmental, health and safety effects

## Abstract

In this contribution, the advantages and limitations of two computational techniques that can be used for the investigation of nanoparticles activity and toxicity: classic nano-QSAR (Quantitative Structure–Activity Relationships employed for nanomaterials) and 3D nano-QSAR (three-dimensional Quantitative Structure–Activity Relationships, such us Comparative Molecular Field Analysis, CoMFA/Comparative Molecular Similarity Indices Analysis, CoMSIA analysis employed for nanomaterials) have been briefly summarized. Both approaches were compared according to the selected criteria, including: efficiency, type of experimental data, class of nanomaterials, time required for calculations and computational cost, difficulties in the interpretation. Taking into account the advantages and limitations of each method, we provide the recommendations for nano-QSAR modellers and QSAR model users to be able to determine a proper and efficient methodology to investigate biological activity of nanoparticles in order to describe the underlying interactions in the most reliable and useful manner.

**Electronic supplementary material:**

The online version of this article (doi:10.1007/s11051-016-3564-1) contains supplementary material, which is available to authorized users.

## Introduction

There has been a significant increase in computational studies related to nanoparticles’ activity and toxicity in the last few years (Ahmed et al. 2013; Durdagi et al. 2008b; Epa et al. 2012; Gajewicz et al. 2015; Mikolajczyk et al. 2015; Puzyn et al. 2011b; Salahinejad 2015; Sizochenko et al. 2014, 2015; Toropov et al. 2012, 2013; Tzoupis et al. 2011; Winkler et al. 2013). The majority of these contributions are based on the main chemistry principle that similar compounds will have similar biological properties (Hansch et al. 1963). The most important group of these techniques is represented by Quantitative Structure–Activity Relationships (QSAR) modelling (Gajewicz et al. 2015; Mikolajczyk et al. 2015; Puzyn et al. 2011b; Salahinejad 2015; Sizochenko et al. 2015; Toropov and Toropova 2015; Toropov et al. 2010; Winkler et al. 2013).

Classical QSAR approach, known also as Hansch Analysis (Hansch et al. 1963), is based on the assumption that biological activity of chemicals is correlated with their physicochemical properties and/or so-called structural descriptors (Puzyn et al. 2010). These descriptors encode certain structural features, such as polarizability, electronic properties and steric parameters. In this case, the developed model includes a set of selected variables (descriptors) that are statistically important and allow providing useful insights and understanding of the mode of studied interaction. However, this approach does not consider the 3D geometric features of the molecules, which leads to some difficulties in adequately describing ligand–receptor interactions. For this type of interactions, better results one can obtained by applying 3D QSAR methodology (Cramer et al. 1988; Klebe et al. 1994; Sippl 2010).

The first application of 3D QSAR technique was proposed in 1988 by Cramer et al. (1988). Their program, the Comparative Molecular Field Analysis (CoMFA) (Cramer et al. 1988), assumes that differences in biological activity correspond to changes in shapes and strengths of non-covalent interaction fields surrounding the molecules (Sippl 2010). Other techniques that also allow to describe 3D interactions in a quantitatively manner include: Comparative Molecular Similarity Indices Analysis (CoMSIA) proposed by Klebe et al. (1994) and the GRID/GOLPE program developed by Reynolds et al. (1989). Both could be considered as the extensions of CoMFA methods that propose to expand its applicability, and in many cases are applied as an alternative to the original CoMFA approach. Taking into account that 3D QSAR techniques consider the ligand properties calculated in its bioactive conformation, it is more suitable than classic approach to study the ligand–receptor interactions (Sippl 2010).

Recently, both classical QSAR and 3D QSAR methodologies are widely applied to study biological activity of nanoparticles (Ahmed et al. 2013; Puzyn et al. 2011b; Tzoupis et al. 2011). Thus, the question: “How to select the best approach in order to properly describe the biological activity of nanomaterials in the most reliable and efficient manner?” may be raised. In this contribution, we compare the efficiency and applicability of both techniques: nano-QSAR (the classic Hansch approach applied for nanomaterials) with 3D nano-QSAR (CoMFA/CoMSIA approach applied for nanomaterials), in order to provide recommendations for QSAR modellers and the models users, to determine the right methodology for investigating nanoparticles’ biological activity.

## Methods

### Nano-QSAR model

#### Objects

Fullerene derivatives were previously studied in order to understand their binding mode to HIV-1 protease based on the 3D-QSAR approaches (Durdagi et al. 2008b; Tzoupis et al. 2011). The CoMFA/CoMSIA models proposed by Tzoupis et al. (2011) were developed for binding energy (BE, kJ/mol) of 74 fullerene derivatives to HIV-1 protease. Among the studied inhibitors, there are 54 compounds, for which BE was calculated with docking simulations and 20 compounds for which the binding energy was obtained experimentally. This is a source of additional variance in the dataset. The average value of the binding energy in the first set (for which BE was calculated with docking simulations) is of an order of magnitude higher than the average binding energy in the second set (for which the binding energy was obtained experimentally).

According to the OECD QSAR recommendations (OECD 2004), data for the modelled property (endpoint) should be obtained with the same methodology/protocol. Thus, theoretically, we would develop classic QSAR model either for the first set of 54 fullerene derivatives or by using the second set of 20 compounds. Tzoupis’s model was calibrated based on the compounds with the computed BE and then validated based on the only 3 compounds with the computed BE and the 20 compounds with the experimentally measured BE. Therefore, we have decided to take the derivatives with the computed BE for calibrating our model.

Thus, the dataset used in this study contains 54 fullerene analogues that were tested for interaction with HIV-1 protease. All data have been taken from the literature (Tzoupis et al. 2011). The structures of the molecules are given in Table 1. Binding energies (BE) for these chemicals to HIV-1 protease have been calculated from docking simulations (Tzoupis et al. 2011).

**Table 1:**
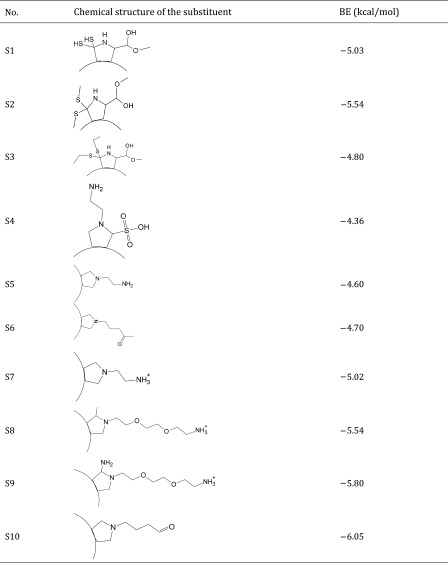

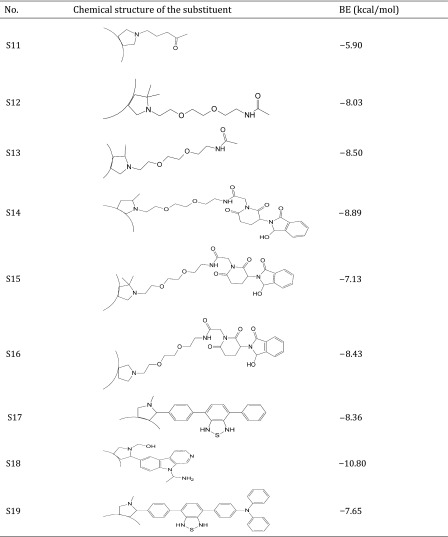

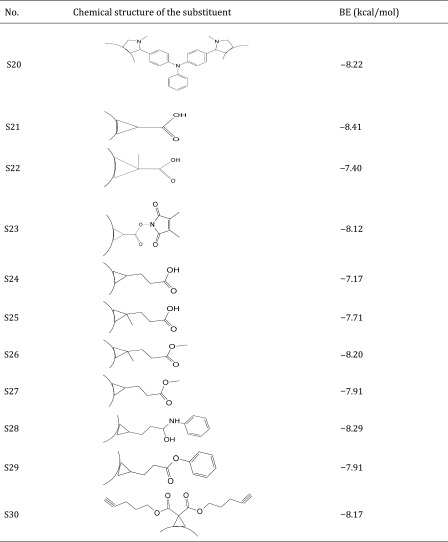

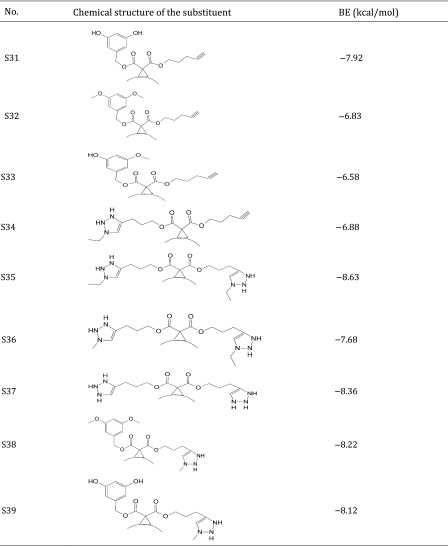

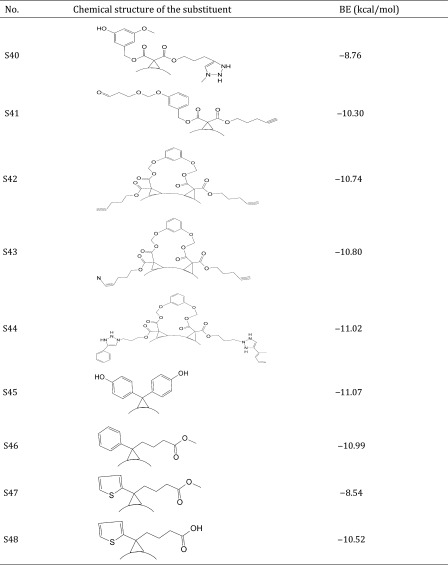

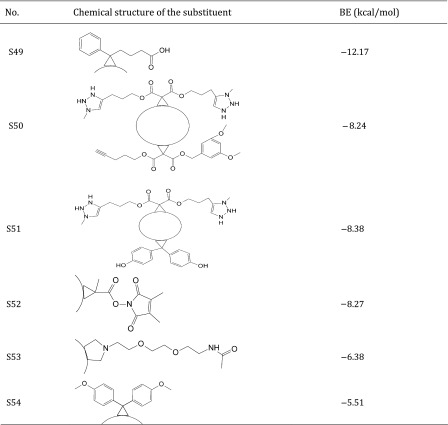
Chemical structures of fullerene derivatives and the values of binding energy (BE) for these carbon-based nanoparticles to HIV-1 protease

Calculated binding energies from docking simulation, values taken from Tzoupis et al. (2011)

#### Quantum–mechanical and descriptors calculations

To obtain optimal geometries of the investigated fullerene derivatives, we applied Density Functional Theory (DFT) approach employing the hybrid meta exchange–correlation functional M06-2X (Zhao and Truhlar 2008). All calculations were performed with the Gaussian 09 code. The 6-31G(d,p) basis set was used throughout the computations (Rassolov et al. 2001).

The following quantum-chemical descriptors were computed for all optimized structures: the total dipole moment, the energy of the highest occupied molecular orbital (E_HOMO_), the energy of the lowest unoccupied molecular orbital (E_LUMO_) and the total energy. In addition, we have calculated structure-based additive descriptors for the fullerene substituents by applying a Dragon (version 6.0) software (Talete 2014). We have assumed, as it was previously described and adopted (Ahmed et al. 2013) that functional groups have a major contribution to the change of the properties of fullerenes since the fullerene core remains constant in each fullerene molecule. Thus, at first, we have calculated a full series of descriptors available in the Dragon software (about 4500 descriptors), then we excluded: (1) descriptors with standard deviation less than 0.0001, (2) descriptors with at least one missing value and (3) descriptors with cross-correlation larger than or equal to *r* = 0.95. The final set of Dragon-generated and quantum–mechanical descriptors contained 1419 descriptors. Then, the whole set of descriptors was used to develop a nano-QSAR (Hansch) model.

#### Nano-QSAR modelling procedure

First, the binding energies of fullerenes to HIV-1 protease were sorted according to the increasing values of the energy (response variable). Then, the dataset was split into two subsets: training and validation ones. The splitting was performed using a 3:1 algorithm according to which, after sorting, every third compound was assigned to a validation set (Puzyn et al. 2011a). Thus, we obtained a training set containing 36 (68 %) compounds and a validation set containing 17 (32 %) of them.

To select the most optimal combination of descriptors, we have used a genetic algorithm (Gramatica et al. 2014) implemented in the QSARINS software (Gramatica et al. 2013) (population size: 300, mutation rate: 65). In addition, descriptors with cross-correlation coefficient values higher than 0.85 have been excluded during final model construction. The chosen descriptors were then used to develop the model using a multiple linear regression analysis (MLR), as it is implemented in QSARINS software (Gramatica et al. 2013). The goodness-of-fit of the developed model was assessed through the squared correlation coefficient (*R* ^2^) and Root-Mean-Square Error of Calibration (RMSE_C_). The Leave-One-Out (LOO) cross-validation technique was utilized for internal validation. The predictivity of the model was assessed by using the squared external validation coefficient (*Q*_Ext_^2^) and Root-Mean-Square Error of Prediction (RMSE_P_) (Gramatica 2007). The applicability domain was verified by applying the Williams plot analysis (Gramatica 2007).

In addition to the above-described internal and external validation tests, an advanced statistical procedure was performed according to recommendation provided by Roy et al. (2015).

### 3D nano-QSAR model

As it was mentioned in the Introduction, the 3D QSAR methodology was recently applied to study the interaction of nanomaterials with their biological targets (Durdagi et al. 2008a, b; Tzoupis et al. 2011). For this contribution, we have adopted CoMFA/CoMSIA approach developed for interaction of fullerene derivatives with HIV-1 protease described in Tzoupis et al. (2011).

### Classic nano-QSAR versus 3D nano-QSAR

Nano-QSAR and 3D nano-QSAR were then compared according to the following criteria: (1) efficiency, which is measured by the obtain statistics, (2) type of experimental data: receptor-base response, cell-based response and tissue-based response, (3) type of nanomaterials: their chemical nature (organic, non-organic, metals) and the homogeneity of their structures, (4) computational cost and time required to perform whole procedure and (5) software availability.

## Results and discussion

### Nano-QSAR model

Based on the values of the binding energy (BE, kcal/mol) and the calculated descriptors, we have developed a classic nano-QSAR model by employing the MLR methodology. The obtained model Eq. (1) is as follows:

1 $${\mathbf{BE}}\left[ {{\text{kcal}}/{\text{mol}}} \right] = - 7.84 + 0.73*{\mathbf{MAXDN}} + 1.77*{\mathbf{GATS2e}} - 0.51*{\mathbf{HNar}} - 0.51*{\mathbf{C}} - {\mathbf{0}}.{\mathbf{07}} - 0.95*{\mathbf{B08}}[{\mathbf{C}} - {\mathbf{O}}]$$

*N*_train_ = 36, *N*_test_ = 17, *R* ^2^ = 0.80, RMSE_c_ = 0.85, *Q*_CV_^2^ = 0.74, RMSE_cv_ = 0.98, *Q*_ext_^2^ = 0.73, RMSE_p_ = 0.92, CCC = 0.86, *r*_m_^2^(training)scaled = 0.80, *r*_maver_^2^(training)scaled = 0.72, Δ*r*_m_^2^(training)scaled = 0.16, *r*_m_^2^(test)scaled = 0.66, *r*_maver_^2^(test)scaled = 0.65, Δ*r*_m_^2^(test)scaled = 0.02, *r*_m_^2^(overall)scaled = 0.76, *r*_maver_^2^(overall)scaled = 0.70, Δ*r*_m_^2^(overall)scaled = 0.12.

As it can be noticed, the developed model represents a linear combination of five descriptors. Two of them belong to topological indices, namely: maximal electrotopological negative variation (**MAXDN**) (Todeschini and Consonni 2009) and Narumi harmonic topological index (**HNar**) (Todeschini and Consonni 2009). Next three descriptors are: Geary autocorrelation of lag 2 weighted by Sanderson electronegativity (**GATS2e**) (Todeschini and Consonni 2009), the descriptor of atom-centred CH2X2 fragment **(C-007)** (Todeschini and Consonni 2009), the presence or absence of C–O at topological distance 8, and **B08**—descriptor that belongs to 2D atom pairs group (Todeschini and Consonni 2009).

The Narumi harmonic topological index (HNar) is related to molecular branching and is proposed as the number of non-hydrogen atoms divided by the reciprocal vertex degree sum (Todeschini and Consonni 2009). According to Eq. 1, higher values of this descriptor decrease the binding energy. The presence of aromatic rings in the structure increases the value of this descriptor. Most probably, the presence of HNar in the model means that the higher number of rings (for example, aromatic cycles) and connected to them functional groups increase the interaction with protein in comparison to structures with the same number of atoms composed of long, linear alkyl chains only. Second topological descriptor, the maximal electrotopological negative variation (MAXDN), describing the maximum negative intrinsic state difference in the molecule is related to the nucleophilicity of the molecule. The positive value (0.726) of the coefficient (Eq. 1) implies that this descriptor is correlated positively with the value of BE. Fullerenes that have more electronegative elements in theirs structures, are characterized by higher values of MAXDN. Next descriptor: Geary autocorrelation of lag 2 weighted by Sanderson electronegativity (GATS2e) represents 2D-autocorrelation classes of descriptors. This descriptor is related to the topology of the structure or its parts that are in association with a given physicochemical property (in this case—Sanderson electronegativity) (Todeschini and Consonni 2009). According to Eq. 1, an increase in GATS2e descriptor’s value increases the binding energy. Another descriptor that is indirectly connected to electronegativity is C-007, which encodes the presence of CH2X2 atom-centred fragment. The X stands for a highly electronegative atom, like oxygen, nitrogen, sulphur, phosphorus and various halogens (Todeschini and Consonni 2009). Only four of the considered structures (S18, S41-S44) display negative value for this descriptor.

Interestingly, we noticed that all descriptors selected by the genetic algorithm into the model (1), although not internally correlated, were connected to the electronegativity to some extent. Certainly, the presence of the electronegative elements results in polarization of a molecule, changes the binding energy, and thereby increases or decreases the interaction between investigated ligands and protein.

**Fig. 1:**
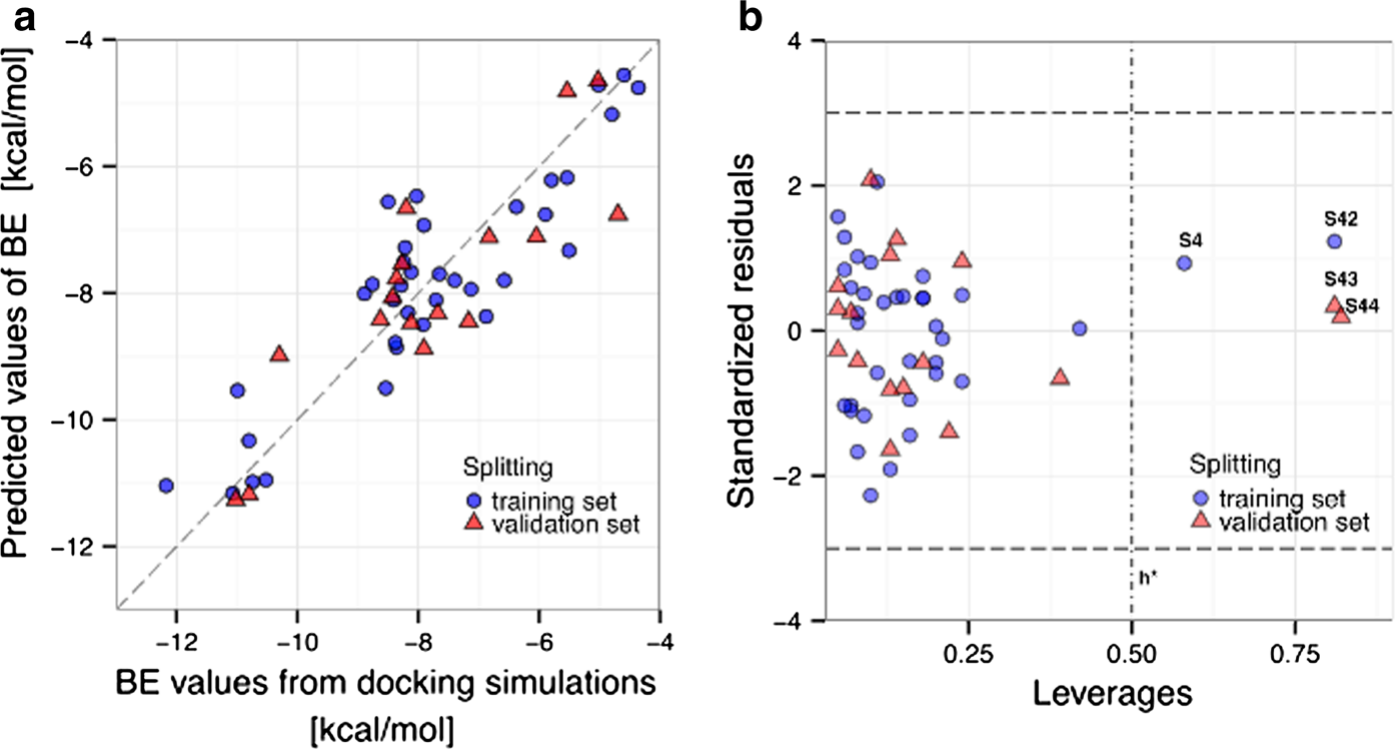
**a** Docking-based versus predicted binding energy plot for the MLR model; **b** Williams plot: standardized residuals versus leverages

Goodness-of-fit, robustness and predictive ability of the developed QSAR model have been confirmed by high values of *R* ^2^, *Q*_CV_^2^, *Q*_Ext_^2^, CCC and relatively low values of the errors represented by: RMSE_C_, RMSE_CV_, RMSE_P_. Moreover, the visual correlation between the observed and predicted values of BE for the training (T) and validation (V) sets confirmed the good quality of the model (Fig. 1a). Additional metrics (summarized under Eq. 1) confirmed the robustness of the developed model as well.

Since the error values (RMSE_C_, RMSE_CV_, RMSE_P_) are at the same level and there are no significantly large residual values for the validation set, one can conclude that the model has not been over fitted (Jagiello et al. 2014). This means that the model predictions are correct not only for the training compounds, but also for external set of compounds.

In order to verify the applicability domain of the nano-QSAR model, we have applied the leverage approach (Gramatica 2007). So-called the Williams plot (Fig. 1b) presents the relationship between leverage values (expressing similarity of a given compound to the training set) and the standardized cross-validated residuals (prediction errors observed for particular compounds). Analysis of the plot confirmed that because the prediction errors for all compounds from the training and validation sets do not exceed the square area between ±3 standard deviation units, there are not outlying predictions observed. The formal leverage (similarity) threshold value h* is equal to 0.51. Interestingly, two compounds from the training set (S4 and S42) and two from validation set (S43 and S44) are characterized by the leverages greater than the threshold value, but—simultaneously—they have small residuals. Such compounds are called “good high leverage points”, and—as it has been previously demonstrated by Jaworska et al. (Jagiello et al. 2014; Jaworska et al. 2005)—compounds having *h*_i_ greater than h*, stabilize the model and make it predictive for new compounds differing structurally from the training set. Obviously, this is the true only when the residuals observed for the training compounds are small.

### 3D nano-QSAR model

Recently, the contributions aimed at the application of 3D QSAR approach for evaluation of the binding energy of fullerene inhibitors to HIV-1 protease have been published (Durdagi et al. 2008a, b; Tzoupis et al. 2011).

In order to compare the classic nano-QSAR (Hansch Analysis) approach with the 3D nano-QSAR, we have adopted contribution proposed by Tzoupis et al. (2011). The main objective of these studies was to design a series of fullerene-based inhibitors for HIV-1 protease by employing the CoMFA/CoMSIA approach. Models proposed by Tzoupis et al. (2011) were developed for the same set of fullerenes as we have used in developing nano-QSAR (Hansch) model (Table 1). Moreover, Tzoupis et al. (2011) obtained models with better statistics than those previously presented by Durdagi et al. (2008a).

### Comparison between the classic nano-QSAR (Hansch Analysis) and 3D nano-QSAR

In order to assess the efficiency of the classic nano-QSAR versus the 3D nano-QSAR, we have made a comparison of the statistics characterizing quality of the predictions for each approach (Table 2). The obtained statistics are very close to each other. Similar statistics were also obtained in previously published contributions related to fullerenes activity against HIV-1 protease (Ahmed et al. 2013; Toropov et al. 2010). Nano-QSAR model developed by Toropov et al. (2010) displays statistics, as follow: *R* ^2^ = 0.9769, *Q*_cv_^2^ = 0.9646, similar to model developed by Ahmed et al. (2013): *R* ^2^ = 0.882, *Q*_cv_^2^ = 0.738. This could suggest that both approaches have similar efficiency and can be applied to study this phenomenon. Which one is more suitable depends on particular task, i.e. a type of information one would like to gather.

**Table 2:** Comparison of statistics obtained in nano-QSAR and 3D nano-QSAR (CoMFA and CoMSIA) approaches

	*R* ^2^	*Q*_cv_^2^	References
nano-QSAR	0.80	0.74	This work
CoMFA	0.84	0.613	Tzoupis et al. (2011)
CoMSIA	0.92	0.763	Tzoupis et al. (2011)

By employing classic nano-QSAR, the certain parts of molecules, that are responsible for the biological activity, have been identified statistically. For example, changes in the electronegativity have been determined to be the driving force of the interactions between fullerenes and HIV-1 protease (this work). CoMFA/CoMSIA analysis of fullerene-based inhibitors of HIV-1 protease pointed out that the highest contribution to the binding energy is associated with the electrostatic interactions, where highly electronegative groups increase (decrease) the affinity of fullerene derivatives to the protein. This is in agreement with the classic nano-QSAR results (Tzoupis et al. 2011). However, the applied 3D QSAR approach indicates also other effects that favour these interactions, such as: hydrophobic interactions and H-bonding (Tzoupis et al. 2011). Thus, one can conclude that classic nano-QSAR allows gathering general knowledge about the mechanism of the studied interaction, while 3D nano-QSAR describes the ligand-based response in more details, relying on their three-dimensional structures.

Additionally, 3D nano-QSAR approach provides a clear visualization, i.e. allows obtaining 3D graphics image superimposed on a core molecule of the dataset. This permits to determine more precisely functional groups of the molecules involved in interactions with residues within the binding pocket of the protease (see details in (Tzoupis et al. 2011)). Such information facilitates appropriate modifications of fullerene derivatives that appropriately improve their binding affinity to the HIV-1 protease.

The literature studies (Kim et al. 1998; Klebe et al. 1994; Podlogar and Ferguson 2000; Puzyn et al. 2010) allow us to provide comprehensive comparison between both techniques. Thus, the advantage of 3D QSAR over Hansch analysis includes also applicability of this approach to evaluate a set of structurally diverse compounds, as long as they act within the same mechanism (Kim et al. 1998; Kubinyi 1998). This advantage, in the case of nanomaterials, is of a high value. Since the classic nano-QSAR is developed with the application of various statistical techniques, they require experimental data measured for sufficient number of considered species. Thus, in order to apply QSAR one needs to have at least 15–20 experimentally measured values of biological activity for chemicals that are located within the applicability domain of the model, which means that they are structurally similar. This principle is often difficult to be fulfilled in case of nanomaterials. Moreover, the application of nano-QSAR is limited also by insufficient set of tools to describe the uniqueness of nanoparticles. More appropriate types of descriptors should reflect not only the molecular structure of these species but also their supra-molecule pattern (e.g. size, shape, porosity, morphology, etc.), and very often their system dependent properties (e.g. agglomeration, formation of protein coronas etc.).

On the other hand, there is no limitation for the application of classic nano-QSAR considering the type of the experimental endpoint values (in vivo and in vitro) and the type of chemicals for which this model could be applied (organic, inorganic, etc.). The various quantum-chemical descriptors can provide useful insight into mode of cytotoxic/toxic action of nanoparticles involving metal oxides (Gajewicz et al. 2015; Toropov et al. 2013), as well as fullerene derivatives (Ahmed et al. 2013; Toropova et al. 2010). Nevertheless, 3D nano-QSAR is much more applicable for organic nanomaterials.

Both techniques require similar calculation time and computational costs. In case of classic nano-QSAR, computer resources are limited mainly by calculations of appropriate nano-descriptors. In 3D QSAR approach, the proper orientation of the ligand to its biological target becomes a crucial factor of success (Kim et al. 1998). The bioactive conformation of ligand can be obtained experimentally, by NMR spectroscopy or X-ray crystallography, or theoretically by means of molecular docking. For both techniques, commercial tools with user-friendly interface are available (Gramatica et al. 2013; TRIPOS Inc. 2001).

In Table 3, we have summarized a comparison of the two considered approaches: nano-QSAR and 3D nano-QSAR according to the described above criteria: type of experimental data; type and characterization of nanomaterials; required software, time and computational costs.

**Table 3:** Applications and requirements of classic nano-QSAR and 3D nano-QSAR

Methods criteria	Nano-QSAR	3D nano-QSAR
Experimental data	Cell-based response, tissue-based response, etc	Ligand-based response
Nanomaterials	Inorganic, organic, metals	Organic
(a) Homogeneity of the chemical structure	homogenous set	Heterogeneous data with the same mode of action
(b) Data preparation	Calculation of nanodescriptors	Knowledge on the bioactive conformation of each molecule (docking)
Statistics obtained	Determination coefficients for calibration and validation, root-mean-square errors	Determination coefficients for calibration and validation, root-mean-square errors
Time	Limited by descriptors’ calculation	Limited by docking procedure
Computational costs	Limited by descriptors’ calculation	Limited by docking procedure
Software	Commercially available in user-friendly software	Commercially available in user-friendly software

### Recommendations

**Fig. 2:**
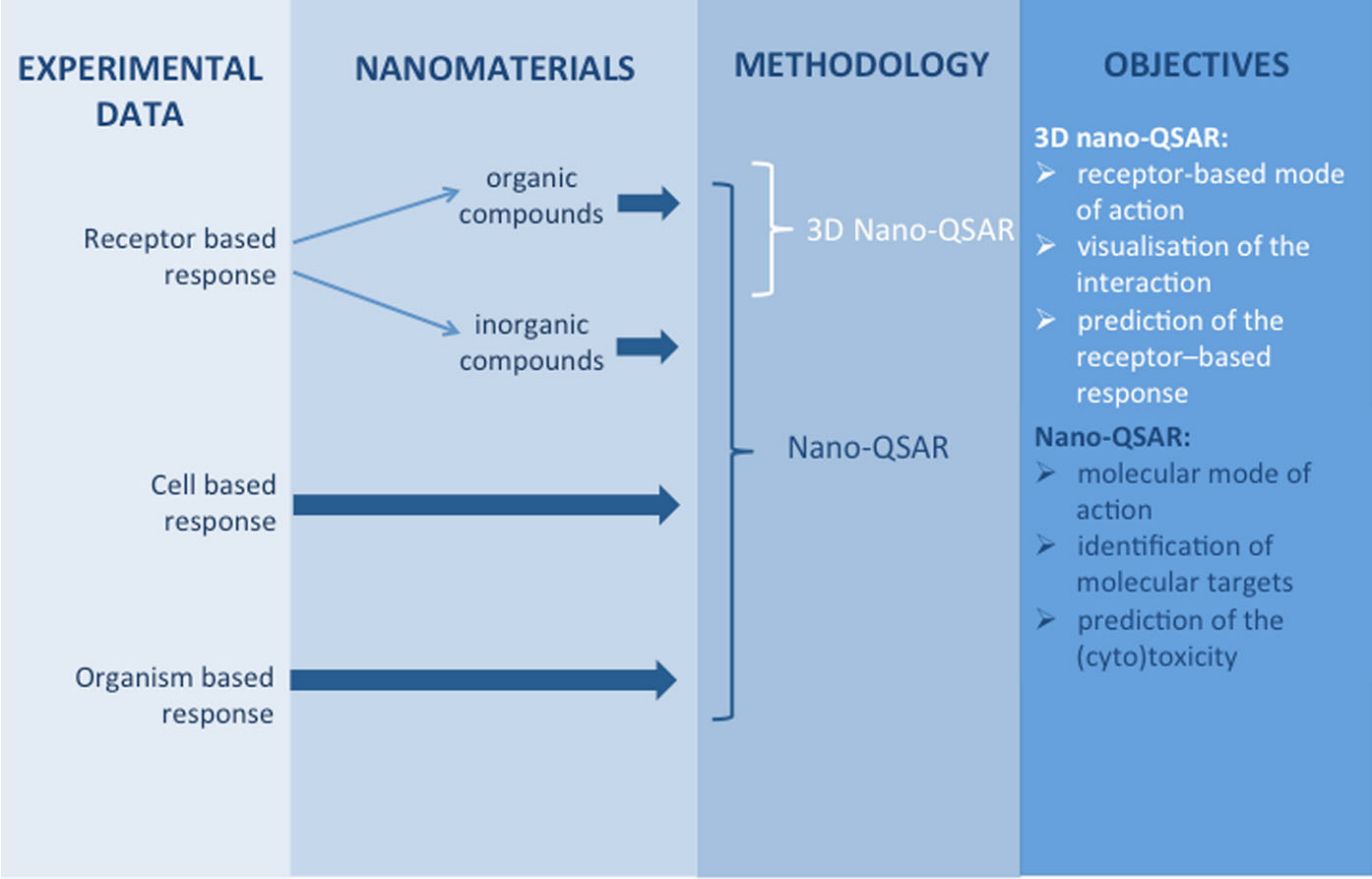
Decision tree for determining application classic or 3D nano-QSAR

Taking into account the advantages and limitations of the nano-QSAR and the 3D nano-QSAR technique, we provide some recommendations for nanomodellers as well as for the users of these methodologies, Fig. 2, to better understand and more efficient predict the biological activity of nanomaterials.

According to the decision tree, shown in Fig. 2, the recommendation which approach should be applied, either classic nano-QSAR or 3D nano-QSAR, in order to better understand the biological activity of nanomaterials, require to answer to the following questions:

1. What types of experimental (response) data are available considered?
2. What types of nanomaterials are considered?
3. What is the major goal of the study?

It is obvious that the adequate experimental data are essential to obtain proper models, both in case of classic nano-QSAR and 3D approach. Appropriate data should fulfil two main principles: (1) should be measured according to the same protocol (ideally if they could be from the same source) and (2) should be symmetrically distributed around their mean value and their precision should be distributed over its range of variation (Kubinyi 1998). The more extensive discussion on biological data for nanomaterials could be found in the literature (Hristozov et al. 2012; Puzyn et al. 2010). Besides the quality of the data, the type of measured response is important to answer the first above-listed question. In this point, it worth to emphasize that classic nano-QSAR represents more universal approach. There are models that have been developed for particular molecular targets response (Ahmed et al. 2013), cell response (Toropov et al. 2013), or the response measured on higher level of organism organization (Toropova et al. 2015). On the other hand, in the 3D nano-QSAR approach the receptor-based response is required. This knowledge one can obtain directly by performing the proper experimental studies (e.g. X-ray crystallography, NMR) or indirectly by applying classic QSAR studies. Defining the type of descriptors that are correlated with the modelled activity, in many cases allows finding the molecular target of the process. Development of 3D nano-QSAR model is not recommended, unless one expects that the analysis will reveal insights into 3D interaction between ligand and receptor in its binding pocket (Puzyn et al. 2010).

The decision on which approach should be applied depends also on the chemical nature of nanomaterials. There is no limitation in application of classic nano-QSAR considering type of chemicals for which this model could be applied (organic, inorganic, metals, etc.). However, 3D nano-QSAR is rather applicable for organic nanomaterials.

The third question refers to the major task of the study. If the biological target is not known, and the objective is to find this target or gather general information about the biological activity of nanomaterials, the classic nano-QSAR would be the right choice. But, if one knows the binding pocket of the studied materials, the 3D nano-QSAR might provide more useful information regarding investigated activity.

## Conclusions

Summing up, we have developed nano-QSAR model allowing to predict the activity of fullerenes derivatives against HIV-1 protease. Developed model was compared with previously published contribution describing the same interaction by means of 3D QSAR approach. Taking into account this case study and literature available studies, the limitations and advantages of each methodology have been discussed. We have developed the recommendation tree for determining a proper methodology to investigate biological activity of nanoparticles. We do believe that both approaches, nano-QSAR and 3D nano-QSAR, could be used simultaneously, if it is possible. Application of classic nano-QSAR model, which is more universal approach, would allow gathering general information about the mode of biological activity of nanomaterials. Then, the 3D QSAR application would help in understanding this activity in detail.

## Electronic supplementary material

Below is the link to the electronic supplementary material.

Supplementary material 1 (DOCX 39 kb)
