# Supplementary material for: Advantages and limitations of classic and 3D QSAR approaches in nano-QSAR studies based on biological activity of fullerene derivatives
Source: J Nanopart Res. 2016 Aug 29;18(9):256. doi: 10.1007/s11051-016-3564-1 (PMC5003910; doi:10.1007/s11051-016-3564-1)
Supplement: Supplementary file 1 — Supplementary material 1 (DOCX 39 kb) [file 11051_2016_3564_MOESM1_ESM.docx]

**Supporting Info.**

**Biological Activity of Fullerene Derivatives – Application of Classic and 3D QSAR Approaches in nano-QSAR studies**

Karolina Jagiello^1^, Monika Grzonkowska^1^, Marta Swirog^1^, Lucky Ahmed^2^, Bakhtiyor Rasulev^2,3^, Aggelos Avramopoulos^4^, Manthos G.Papadopoulos^4^, Jerzy Leszczynski^2^, and Tomasz Puzyn^1*^

*^1^ Laboratory of Environmental Chemometrics, Institute for Environmental and Human Health Protection, Faculty of Chemistry, University of Gdansk, Wita Stwosza 63, 80-308 Gdansk, Poland*

*^2^ Interdisciplinary Nanotoxicity Center, Department of Chemistry and Biochemistry, Jackson State University, 1400 JR Lynch Street, Jackson MS 39217-0510, USA*

*^3^ Center for Computationally Assisted Science and Technology, North Dakota State University, 1805 NDSU Research Park Drive, Post Office Box 6050, Fargo, North Dakota, 58108, USA*

*^4^ Institute of Biology, Pharmaceutical Chemistry and Biotechnology, National Hellenic Research Foundation, 48 Vas. Constantinou Ave., Athens 11635, Greece*

***Corresponding author**: **Prof. Tomasz Puzyn**,
Laboratory of Environmental Chemometrics, Faculty of Chemistry, University of Gdańsk,
Wita Stwosza 63, 80-308 Gdansk, Poland.
Tel.: +48 58 523 52 48; E-mail address: [t.puzyn@qsar.eu.org](mailto:t.puzyn@qsar.eu.org)

**Table ES1. Dataset splitting for QSAR model**

BE^obs^ [kcal/mol] -calculated binding energy from docking simulations

BE^pred^ [kcal/mol] -predicted binding energy from QSAR model

T - training set

V - validation set

| **No.** | **Name** | **Set splitting** | **MAXDN** | **GATS2e** | **HNar** | **C-007** | **B08[C-O]** | **BE^obs^ [kcal/mol]** | **BE^pred^ [kcal/mol]** | **Standardized residuals** | **Leverages** |
| --- | --- | --- | --- | --- | --- | --- | --- | --- | --- | --- | --- |
| 1 | S1 | V | 2.087 | 1.058 | 1.590 | 0 | 0 | -5.03 | -4.65 | -0.44 | 0.18 |
| 2 | S2 | V | 2.013 | 1.078 | 1.642 | 0 | 0 | -5.54 | -4.82 | -0.79 | 0.15 |
| 3 | S3 | T | 2.012 | 1.036 | 1.682 | 0 | 0 | -4.80 | -5.18 | 0.39 | 0.12 |
| 4 | S4 | T | 4.820 | 0.533 | 1.618 | 0 | 0 | -4.36 | -4.76 | 0.93 | 0.58 |
| 5 | S5 | T | 0.681 | 1.555 | 1.846 | 0 | 0 | -4.60 | -4.56 | -0.11 | 0.21 |
| 6 | S6 | V | 1.343 | 0.912 | 1.784 | 0 | 0 | -4.70 | -6.76 | 2.08 | 0.10 |
| 7 | S7 | T | 0.644 | 1.528 | 1.846 | 0 | 0 | -5.02 | -4.72 | -0.44 | 0.20 |
| 8 | S8 | T | 0.801 | 1.603 | 1.837 | 0 | 1 | -5.54 | -6.18 | 0.75 | 0.18 |
| 9 | S9 | T | 1.087 | 1.539 | 1.837 | 0 | 1 | -5.80 | -6.22 | 0.46 | 0.14 |
| 10 | S10 | V | 0.990 | 0.980 | 1.875 | 0 | 0 | -6.05 | -7.11 | 1.04 | 0.13 |
| 11 | S11 | T | 1.343 | 0.912 | 1.784 | 0 | 0 | -5.90 | -6.76 | 0.94 | 0.10 |
| 12 | S12 | T | 1.687 | 1.294 | 1.740 | 0 | 1 | -8.03 | -6.47 | -1.91 | 0.13 |
| 13 | S13 | T | 1.686 | 1.323 | 1.800 | 0 | 1 | -8.50 | -6.56 | -2.27 | 0.10 |
| 14 | S14 | T | 2.605 | 0.919 | 1.897 | 0 | 1 | -8.89 | -8.01 | -1.03 | 0.07 |
| 15 | S15 | T | 2.607 | 0.904 | 1.861 | 0 | 1 | -7.13 | -7.94 | 0.84 | 0.06 |
| 16 | S16 | V | 2.604 | 0.932 | 1.929 | 0 | 1 | -8.43 | -8.07 | -0.42 | 0.08 |
| 17 | S17 | V | 0.763 | 1.115 | 2.160 | 0 | 0 | -8.36 | -7.77 | -0.66 | 0.39 |
| 18 | S18 | T | 1.439 | 1.029 | 2.000 | 1 | 1 | -10.80 | -10.33 | -0.70 | 0.24 |
| 19 | S19 | T | 0.792 | 1.143 | 2.182 | 0 | 0 | -7.65 | -7.70 | 0.03 | 0.42 |
| 20 | S21 | T | 2.296 | 0.325 | 1.636 | 0 | 0 | -8.41 | -8.11 | -0.42 | 0.16 |
| 21 | S22 | T | 2.313 | 0.299 | 1.527 | 0 | 0 | -7.40 | -7.80 | 0.49 | 0.24 |
| 22 | S23 | T | 2.202 | 0.487 | 1.698 | 0 | 0 | -8.12 | -7.67 | -0.58 | 0.11 |
| 23 | S24 | V | 2.322 | 0.312 | 1.714 | 0 | 0 | -7.17 | -8.45 | 1.26 | 0.14 |
| 24 | S25 | T | 2.330 | 0.299 | 1.612 | 0 | 0 | -7.71 | -8.11 | 0.45 | 0.18 |
| 25 | S26 | V | 1.742 | 0.743 | 1.644 | 0 | 0 | -8.20 | -6.66 | -1.64 | 0.13 |
| 26 | S27 | T | 1.734 | 0.768 | 1.742 | 0 | 0 | -7.91 | -6.93 | -1.17 | 0.09 |
| 27 | S28 | T | 1.724 | 0.778 | 2.000 | 0 | 0 | -8.29 | -7.88 | -0.59 | 0.20 |
| 28 | S29 | V | 1.775 | 0.557 | 2.000 | 0 | 0 | -7.91 | -8.88 | 0.95 | 0.24 |
| 29 | S30 | T | 2.313 | 0.784 | 1.740 | 0 | 1 | -8.17 | -8.31 | 0.11 | 0.08 |
| 30 | S31 | T | 2.463 | 0.748 | 1.781 | 0 | 1 | -7.92 | -8.50 | 0.59 | 0.07 |
| 31 | S32 | V | 2.404 | 1.063 | 1.796 | 0 | 1 | -6.83 | -7.12 | 0.25 | 0.07 |
| 32 | S33 | T | 2.433 | 0.909 | 1.789 | 0 | 1 | -6.58 | -7.80 | 1.29 | 0.06 |
| 33 | S34 | T | 2.318 | 0.846 | 1.834 | 0 | 1 | -6.88 | -8.37 | 1.57 | 0.05 |
| 34 | S35 | V | 2.323 | 0.891 | 1.902 | 0 | 1 | -8.63 | -8.42 | -0.27 | 0.05 |
| 35 | S36 | V | 2.321 | 0.908 | 1.898 | 0 | 1 | -7.68 | -8.32 | 0.61 | 0.05 |
| 36 | S37 | T | 2.317 | 0.868 | 1.987 | 0 | 1 | -8.36 | -8.86 | 0.51 | 0.09 |
| 37 | S38 | T | 2.407 | 1.083 | 1.861 | 0 | 1 | -8.22 | -7.28 | -1.10 | 0.07 |
| 38 | S39 | V | 2.467 | 0.812 | 1.851 | 0 | 1 | -8.12 | -8.47 | 0.30 | 0.05 |
| 39 | S40 | T | 2.437 | 0.949 | 1.856 | 0 | 1 | -8.76 | -7.86 | -1.03 | 0.06 |
| 40 | S41 | V | 2.424 | 1.001 | 1.851 | 1 | 1 | -10.30 | -8.99 | -1.40 | 0.22 |
| 41 | S42 | T | 2.526 | 0.857 | 1.854 | 2 | 1 | -10.74 | -10.98 | 1.23 | 0.81 |
| 42 | S43 | V | 2.528 | 0.813 | 1.854 | 2 | 1 | -10.80 | -11.18 | 0.34 | 0.81 |
| 43 | S44 | V | 2.558 | 0.881 | 1.964 | 2 | 1 | -11.02 | -11.26 | 0.19 | 0.82 |
| 44 | S45 | T | 1.366 | 0.560 | 1.981 | 0 | 1 | -11.07 | -11.16 | 0.06 | 0.20 |
| 45 | S46 | T | 1.760 | 0.761 | 1.901 | 0 | 1 | -10.99 | -9.54 | -1.67 | 0.08 |
| 46 | S47 | T | 1.753 | 0.766 | 1.895 | 0 | 1 | -8.54 | -9.50 | 1.02 | 0.08 |
| 47 | S48 | T | 2.341 | 0.341 | 1.888 | 0 | 1 | -10.52 | -10.95 | 0.47 | 0.15 |
| 48 | S49 | T | 2.348 | 0.327 | 1.895 | 0 | 1 | -12.17 | -11.04 | -1.44 | 0.16 |
| 49 | S50 | T | 3.020 | 0.987 | 1.948 | 0 | 1 | -8.24 | -7.51 | -0.95 | 0.16 |
| 50 | S51 | T | 2.856 | 0.822 | 2.038 | 0 | 1 | -8.38 | -8.78 | 0.45 | 0.18 |
| 51 | S52 | V | 2.208 | 0.467 | 1.641 | 0 | 0 | -8.27 | -7.54 | -0.81 | 0.13 |
| 52 | S53 | T | 1.685 | 1.351 | 1.855 | 0 | 1 | -6.38 | -6.64 | 0.24 | 0.08 |
| 53 | S54 | T | 1.043 | 1.432 | 1.983 | 0 | 1 | -5.51 | -7.33 | 2.05 | 0.11 |
